# Knowledge and Practice of Maternal Vaccination during Pregnancy: A Cross-sectional Survey of Selected Obstetricians

**DOI:** 10.4314/ejhs.v34i6.3

**Published:** 2024-11

**Authors:** Akaninyene Eseme Ubom, Elif Goknur Topcu, Elhadi Miskeen, Olire Christine Afon, Priyankur Roy, Francisco Ruiloba, David M Aqua, Emmanuel E John

**Affiliations:** 1 Department of Obstetrics, Gynaecology and Perinatology, Obafemi Awolowo University Teaching Hospitals Complex, Ile-Ife, Nigeria; 2 World Association of Trainees in Obstetrics and Gynecology (WATOG), Paris, France; 3 Department of Obstetrics and Gynaecology, Assisted Reproductive Technologies Unit, Acibadem Maslak Hospital, Istanbul, Turkey; 4 Department of Obstetrics and Gynecology, College of Medicine, University of Bisha, Saudi Arabia; 5 Roy's Multispecialty Hospital, Siliguri, India; 6 Fetal Medicine Research Institute, King's College Hospital, UK; 7 Department of Obstetrics and Gynaecology, University of Nigeria Teaching Hospital, Enugu, Nigeria; 8 Department of Obstetrics and Gynaecology, University of Uyo Teaching Hospital, Uyo, Nigeria

**Keywords:** Maternal vaccination, vaccine safety, global health, low-and-middle income countries (LMICs)

## Abstract

**Background:**

Maternal vaccination is a key strategy to meet Sustainable Development Goal 3, aiming to eliminate preventable deaths among newborns and children under five by 2030. This study explored obstetricians' knowledge and practices regarding maternal vaccination globally.

**Methods:**

A cross-sectional survey targeting obstetricians was conducted by the World Association of Trainees in Obstetrics and Gynaecology (WATOG,) from June 25 to July 18, 2023. A structured 25-item questionnaire, distributed electronically, gathered data on sociodemographics and vaccination knowledge and practices. Data analysis was performed using IBM Statistical Product and Service Solutions (SPSS) Statistics for Windows, version 24.

**Results:**

Ninety-two obstetricians participated. Only 16.3% exhibited good knowledge of safe vaccines in pregnancy. Fear of teratogenicity was the primary reason (58.7%) for vaccine hesitancy among pregnant women. Approximately 47.8% of participants indicated that only tetanus toxoid was routinely available in their hospitals, with 48.9% reporting that women had to pay for vaccines. Nonetheless, 62% stated their countries had national vaccination guidelines for pregnant women.

**Conclusion:**

The study identifies significant gaps in obstetricians' knowledge of vaccine safety, alongside barriers related to availability and cost, impacting maternal vaccination uptake.

## Introduction

Vaccination is a pivotal public health measure that has significantly reduced the incidence of various diseases (1). Historical data demonstrates that vaccination has led to the near eradication of diseases like smallpox and polio, showcasing its profound impact on global health. Recent evidence during the COVID-19 pandemic highlights that vaccination can prevent millions of deaths (2). The economic advantages of vaccination are significant, particularly in low-and-middle income countries (LMICs), where the return on investment in immunization programs can be substantial (3).

Maternal vaccination plays a critical role in safeguarding the health of mothers and their newborns against infectious diseases. Given the unique immunological changes during pregnancy, women become more susceptible to infections (4). Vaccines administered during pregnancy can provide passive immunity to newborns, crucial in regions where neonatal infections are prevalent (5-7).

Despite the known benefits, vaccine uptake in pregnancy is alarmingly low in many areas, primarily due to hesitancy stemming from concerns about safety and misinformation (8,9). Healthcare provider (HCP) recommendations are vital in influencing vaccine acceptance among pregnant women (10-12). Therefore, understanding HCPs' knowledge and attitudes towards maternal vaccination is essential.

This study assessed the knowledge and practices regarding maternal vaccination among obstetricians across diverse global settings.

## Materials and Methods

**Study design and population**: This cross-sectional survey involved selected obstetrics and gynecology (obgyn) trainees and specialists from Africa, Asia, Europe, North America, South America, and Oceania. The study was conducted over three weeks, from June 25 to July 18, 2023. It was organized by the World Association of Trainees in Obstetrics and Gynaecology (WATOG), an affiliate of the International Federation of Gynecology and Obstetrics (FIGO).

Participants included 60 obgyn trainees and young specialists chosen globally by FIGO to take part in the 2023 FIGO One World Exchange (OWE), a clinical exchange program held in Paris, France, in October 2023. These OWE Fellows participated in clinical observerships in French hospitals and subsequently attended the XXIV FIGO World Congress of Gynecology and Obstetrics in Paris. Additional participants were recruited through convenience sampling from the FIGO and WATOG networks.

**Sample size determination**: The sample size for this study was calculated using the formula for descriptive studies (13). The proportion of HCPs expected to have poor knowledge, attitude and practice of maternal vaccination was estimated at 5% from a previous study by Healy *et al.* (14), giving an approximate sample size of 73. Allowing for a 10% attrition rate, the minimum required sample size was calculated to be 80.

**Survey instrument**: A 25-item structured questionnaire was designed using Google Forms®. The questionnaire collected information on the sociodemographic characteristics of participants, as well as their knowledge and practices regarding maternal vaccination during pregnancy. Most questions were multiple-choice, with a few, open-ended.

Questions assessing knowledge of safe vaccines in pregnancy were formatted as multiple-choice items. Respondents earned one point for each correct answer, while incorrect responses received zero points. Knowledge levels were categorized as follows:

“Good/Adequate” knowledge for scores of 80-100% correct,

“Average” knowledge for scores of 60-79% correct,

“Poor” knowledge for scores below 60%, based on Bloom's cutoffs (15).

The questionnaire was pretested on 15 OWE Fellows to ensure reliability and validity before final distribution.

**Data collection**: The questionnaire was electronically distributed to study participants. Weekly reminders were sent to those who had not responded. The survey was completely anonymized, with no identifying information collected from respondents. To maintain the integrity of the survey, the Google Form® was configured to allow only one response per participant. By completing and submitting the survey, participants provided implied consent, which was indicated at the beginning of the questionnaire. Participation was voluntary, and no incentives were offered.

**Statistical analysis**: Data were analyzed using IBM Statistical Product and Service Solutions (SPSS) Statistics for Windows, version 24 (IBM Corp., Armonk, NY, USA). Results were presented in tables and percentages. Associations between good knowledge of safe vaccines during pregnancy and sociodemographic characteristics were tested using Pearson's Chi-square test. A P-value of less than 0.05 was considered statistically significant.

## Results

Ninety-two obstetricians from 31 countries participated, with majority being females (62, 67.4%) and practicing in LMICs (78/90, 86.7%) (Table 1).

**Table 1:** Sociodemographic characteristics of the study participants (n=92)

Characteristic	Frequency (%)
**Age**	
20-29	13 (14.1)
30-39	60 (65.2)
≥40	19 (20.7)
**Gender**	
Male	30 (32.6)
Female	62 (67.4)
**Marital status**	
Single	29 (31.5)
Married	62 (67.4)
Divorced	1 (1.1)
**Cadre**	
Senior House Officer	2 (2.2)
Registrar/Junior Resident	28 (30.4)
Senior/Specialist Registrar	46 (50.0)
Consultant/Attending	16 (17.4)
**Type of hospital**	
Primary Health Centre (Government)	1 (1.1)
General Hospital/Secondary Level Hospital (Government)	11 (12.0)
Tertiary Level Hospital (Government)	71 (77.2)
Private Hospital	9 (9.8)
**Country of practice (n=90)**	
High income	12 (13.3)
Middle income	70 (77.8)
Low income	8 (8.9)
**Region of practice**	
Africa	51 (55.4)
Asia	16 (17.4)
Europe	13 (14.1)
North America	4 (4.3)
South America	6 (6.5)
Oceania	2 (2.2)

**Knowledge of vaccine safety in pregnancy**: One-tenth of the study respondents (9, 9.8%) were either indifferent (2, 2.2%) or disagreed/strongly disagreed (7, 7.6%) that vaccines should be administered during pregnancy. All participants in the latter category cited teratogenic concerns, while 71.4% (5) feared potential adverse maternal effects, such as miscarriage (Table 2).

**Table 2:** Knowledge and attitudes of vaccine safety in pregnancy amongst study participants (n=92)

Characteristic	Frequency (%)
**Pregnant women should be administered vaccines**	
Strongly agree	44 (47.8)
Agree	39 (42.4)
Disagree	5 (5.4)
Strongly disagree	2 (2.2)
Indifferent	2 (2.2)
**Reason for Disagreeing/Strongly Disagreeing (n=7)** *	
Teratogenic concerns	7 (100.0)
Possible adverse maternal effects in pregnancy (eg miscarriage)	5 (71.4)
Vaccines are not useful in pregnancy	3 (42.9)
Cost	1 (14.3)
**Knowledge of safe vaccines in pregnancy**	
Good/Adequate	15 (16.3)
Average	39 (42.4)
Poor	38 (41.3)
**Is COVID-19 vaccine safe in pregnancy?**	
Yes	65 (70.7)
No	10 (10.9)
I don't know	17 (18.5)
**Safest trimester to administer vaccines in pregnancy**	
First	5 (5.4)
Second	66 (71.7)
Third	21 (22.8)

* multiple responses from each respondent, hence n>7

Less than one-fifth of the participants (15, 16.3%) demonstrated good/adequate knowledge of safe vaccines in pregnancy. Bivariate analysis revealed no association between good knowledge and sociodemographic characteristics, including age (P=0.215), gender (P=0.504), marital status (P=0.688), professional cadre (P=0.706), type of hospital (P=0.711), and country of practice (P=0.689) (Table 3). Nearly one-third (27, 29.3%) were unaware that COVID-19 vaccines could be safely administered during pregnancy. However, most respondents (66, 71.7%) agreed that the second trimester is the safest time to administer vaccines during pregnancy.

**Table 3:** Association between knowledge of safe vaccines in pregnancy and sociodemographic characteristics of the study participants

Characteristic	Good knowledge of safe vaccines		*p*-value

	Yes (%)	No (%)	
**Age (years)**			
<30	4 (30.8)	9 (69.2)	0.215
≥30	11 (13.9)	68 (86.1)	
**Gender**			
Male	6 (20.0)	24 (80.0)	0.504
Female	9 (14.5)	53 (85.5)	
**Marital status**			
Married	9 (14.5)	53 (85.5)	0.688
Single	6 (20.7)	23 (79.3)	
Divorced	0 (0)	1 (100.0)	
**Cadre**			
Senior house officer	0 (0)	2 (100.0)	0.706
Registrar/Junior Resident	4 (14.3)	24 (85.7)	
Senior/Specialist Registrar	7 (15.2)	39 (84.8)	
Consultant/Attending	4 (25.0)	12 (75.0)	
**Type of hospital**			
Primary Health Centre (Government)	0 (0)	1 (100.0)	0.711
General Hospital/Secondary level hospital (Government)	3 (27.3)	8 (72.7)	
Tertiary level hospital (Government)	11 (15.5)	60 (84.5)	
Private hospital	1 (11.1)	8 (88.9)	
**Country of practice**			
High income country	3 (25.0)	9 (75.0)	0.689
Middle income country	11 (15.7)	59 (84.3)	
Low income country	1 (12.5)	7 (87.5)	

**Practice of maternal vaccination during pregnancy**: The most commonly available vaccines during pregnancy were tetanus toxoid (TT) (69, 75.0%), COVID-19 (24, 26.1%), whooping cough/pertussis/tetanus, diphtheria, and acellular pertussis (Tdap) (23, 25.0%), and flu vaccines (19, 20.7%). Almost half of the respondents (44, 47.8%) stated that only TT was available for administration in their facilities, all of which were located in low- and middle-income countries (LMICs). Three respondents (3.3%) reported that no maternal vaccines were available in their hospitals (Table 4).

**Table 4:** Practice of maternal vaccination during pregnancy (n=92)

Characteristic	Frequency	Percent
**Available/routinely administered vaccines in pregnancy in your hospital** *		
Tetanus toxoid	69	75.0
COVID-19	24	26.1
Whooping cough/pertussis/Tdap^†^	23	25.0
Flu	19	20.7
Hepatitis B	10	10.9
Diphtheria	4	4.3
Others	3	3.3
None	3	3.3
**Vaccine cost**		
All available vaccines are free	47	51.1
All available vaccines are free but the women pay for consumables such as gloves and syringes for vaccine administration	18	19.6
Available vaccines are paid for by the women	27	29.3
**Who counsels pregnant women for vaccination in your hospital?**		
The obstetrician	24	26.1
The midwife/antenatal care nurse	10	10.9
Both the obstetrician and midwife/antenatal care nurse	57	62.0
Primary health/infectious diseases physician	1	1.1
**Commonest reason why pregnant women decline vaccines in your hospital**		
Fear of teratogenicity	54	58.7
Fear of harmful effect on pregnancy	18	19.6
Others	5	5.4
No reason/I don't know	15	16.3
**What do you do when pregnant women decline vaccines?** *		
I re-counsel them at subsequent visits	69	75.0
I ask another HCP to re-counsel them	20	21.7
I respect their decision and do nothing	17	18.5
**Does your country have a national guideline on vaccination in pregnancy?**		
Yes	57	62.0
No	15	16.3
I don't know	20	21.7

* Multiple responses from the respondents, hence n>92

† Tdap=Tetanus, diphtheria, and acellular pertussis

Nearly half of the participants (45, 48.9%) indicated that pregnant women either had to pay for vaccines (27, 29.3%) or for consumables used in vaccination (18, 19.6%). In most cases, pregnant women were counseled about vaccination by both obstetricians and antenatal care nurses/midwives (57, 62.0%). The predominant reason for declining vaccines was fear of teratogenicity (54, 58.7%). When women declined vaccination, most respondents re-counseled them at subsequent visits (69, 75.0%) or asked another healthcare provider to do so (20, 21.7%) (Table 4).

More than one-third of participants (35, 38.0%) reported that their countries lacked national guidelines on maternal immunization during pregnancy. Specifically, 15 (16.3%) were aware that no guidelines existed, while 20 (21.7%) did not know. All those from high-income countries (HICs) (12/12, 100%) reported national vaccination guidelines, whereas only 43 out of 78 of those from LMICs (55.1%) reported such guidelines. This difference was statistically significant (P=0.035).

## Discussion

It is recommended that all pregnant women receive Tdap, flu, and COVID-19 vaccines; other vaccines should be administered based on risk versus benefit considerations (16,17). In our study, these vaccines were the most commonly available and administered during pregnancy. Among the routinely recommended vaccines, only tetanus has been implemented globally (10). Tetanus toxoid was the most commonly available vaccine, administered in 75% of hospitals in our study. Through the World Health Organization (WHO) Maternal Neonatal Tetanus Elimination (MNTE) initiative, 47 of 59 priority countries had achieved tetanus elimination as of December 2020 (defined as fewer than one case per 1,000 live births per year), with a 96% reduction in annual tetanus-related neonatal deaths—from an estimated 780,000 in 1988 to 34,000 in 2015 (18,19).

Despite WHO recommendations (endorsed by FIGO) and robust evidence supporting the safety of both influenza and pertussis vaccines during pregnancy, persistent safety concerns among consumers limit the uptake of these vaccines (10). In our study, only 25% and 21% of hospitals routinely administered pertussis/Tdap and flu vaccines, respectively. In England, vaccine uptake rates for the flu vaccine during pregnancy have been reported at 45% (20). Aside from safety concerns, challenges in implementing maternal influenza immunization programs in tropical and subtropical LMICs include the alignment of current influenza vaccine formulations and expiry dates with Northern and Southern Hemisphere influenza seasons, while tropical and subtropical countries often face year-round disease (21).

COVID-19 vaccines are considered safe during pregnancy and are recommended by several health organizations, including the Centers for Disease Control, the American College of Obstetricians and Gynecologists, the American Society for Reproductive Medicine, and the Society for Maternal Fetal Medicine (22,23). According to the WHO, although many countries are making efforts to integrate COVID-19 vaccination into routine immunization and primary healthcare services, inequalities in vaccination coverage still exist, disproportionately affecting the lowest-income countries (24). In our study, only 26% of respondents, primarily from LMICs, reported administering the COVID-19 vaccine to pregnant women in their hospitals. Pregnant women exhibit significantly higher vaccine hesitancy for COVID-19 compared to non-pregnant women (25,26). A significant motivating factor for pregnant women to receive the COVID-19 vaccine (and other vaccines) is an HCP's recommendation (27). The influence of HCP recommendations on vaccine uptake among pregnant women has also been highlighted by other authors (14,28).

The knowledge of HCPs regarding the safety of the COVID-19 vaccine and other vaccines in pregnancy affects their willingness to recommend these vaccines to pregnant women. Almost 30% of respondents in our study did not recognize that the COVID-19 vaccine could be safely administered during pregnancy. Furthermore, less than one-fifth demonstrated good/adequate knowledge of vaccines safe for use during pregnancy, with less than half strongly agreeing that pregnant women should receive vaccinations. Many HCPs lack up-to-date knowledge and tend to exaggerate the potential side effects of vaccines during pregnancy (29). A cross-sectional study in Italy found that over 30% of HCPs were unaware that the Tdap vaccine should only be administered in the third trimester of pregnancy (30). Adequate training for HCPs on infectious diseases in pregnancy, as well as the vaccines for these diseases and their safety, is essential. Well-informed HCPs can effectively address the fears, concerns, misconceptions, and misinformation that pregnant women may have regarding vaccines and provide education on their efficacy and safety. This is particularly important, as many pregnant women decline vaccinations due to concerns about their babies' safety (10,27), as reported by nearly 60% of our study respondents. To equip HCPs to provide accurate vaccine information, targeted educational programs are necessary to enhance their knowledge of maternal vaccination (31).

While all HICs in our study had national guidelines on vaccination during pregnancy, just over 50% of LMICs had such guidelines. Almost half of our participants reported that pregnant women in their facilities had to pay for vaccines or the consumables used for administration. The lack of guidelines and the costs associated with vaccines, among other challenges, continue to hinder vaccine availability, especially in low-resource settings (8,21). In nearly half of the hospitals in LMICs participating in our study, TT was the only routine vaccine available during pregnancy. To enhance vaccine availability and uptake, it is crucial to develop guidelines and protocols for immunization during pregnancy and to reduce immunization costs (8).

This study has limitations, including its descriptive nature and reliance on convenience sampling, which may introduce selection bias. Additionally, the small sample size limits the generalizability of our findings. The study is also subject to information and recall biases, as the reported data relied on respondents' recollections of maternal vaccination practices in their countries, with over 85% of respondents from LMICs, where knowledge of vaccination during pregnancy was poorer compared to HICs. Despite these limitations, the study offers valuable groundwork for more extensive research and important data to inform interventions aimed at improving the practice of vaccination during pregnancy.

In conclusion, the insights gained from this study have significant implications for policy formulation and clinical practice protocols aimed at increasing vaccine uptake during pregnancy, with the goal of achieving the SDG 3 target of ending all preventable neonatal and under-five mortality by 2030. Healthcare providers play vital roles in providing information, addressing women's questions, recommending vaccines, and administering vaccinations within routine maternity care. These roles can be effectively performed only if HCPs are well-trained and knowledgeable about maternal immunization. Less than one-fifth of HCPs in our study demonstrated good knowledge of vaccine safety in pregnancy. There is a pressing need for regular training and retraining of HCPs on maternal vaccination. Other interventions to potentially increase vaccine availability and uptake in pregnancy include formulating national guidelines and protocols, as well as reducing vaccine costs, which our study identified as ongoing challenges in many settings, particularly in LMICs.

## References

[1] Orenstein WA, Ahmed R (2017). Simply put: Vaccination saves lives. Proc Natl Acad Sci USA.

[2] Watson OJ, Barnsley G, Toor J, Hogan AB, Winskill P, Ghani AC (2022). Global impact of the first year of COVID-19 vaccination: a mathematical modelling study. Lancet Infect Dis.

[3] Ozawa S, Clark S, Portnoy A, Grewal S, Brenzel L, Walker DG (2016). Return on investment from childhood immunization in low- and middle-income countries, 2011-20. Health Aff.

[4] Mackin DW, Walker SP (2021). The historical aspects of vaccination in pregnancy. Best Pract Res Clin Obstet Gynaecol.

[5] Sobanjo-ter Meulen A, Abramson J, Mason E, Rees H, Schwalbe N, Bergquist S (2015). Path to impact: a report from the Bill and Melinda Gates Foundation convening on maternal immunization in resource-limited settings; Berlin–January 29–30, 2015. Vaccine.

[6] Kirkby K, Bergen N, Baptista A, Schlotheuber A, Hosseinpoor AR (2023). Data Resource Profile: World Health Organization Health Inequality Data Repository. Int J Epidemiol.

[7] Roser M (2023). Ensure healthy lives and promote well-being for all at all ages. Our World in Data.

[8] Amaral E, Cain JM, Hearing F, Lumsden MA (2023). FIGO guidance for sustainable implementation of vaccination programs for women: Pregnancy and HPV. Int J Gynecol Obstet.

[9] Wilson R, Paterson P, Larson HJ (2019). Strategies to improve maternal vaccination acceptance. BMC Public Health.

[10] Krishnaswamy S, Lambach P, Giles ML (2019). Key considerations for successful implementation of maternal immunization programs in low and middle income countries. Hum Vaccin Immunother.

[11] Licata F, Romeo M, Riillo C, Di Gennaro G, Bianco A (2023). Acceptance of recommended vaccinations during pregnancy: a cross-sectional study in Southern Italy. Front Public Health.

[12] Gianfredi V, Berti A, Stefanizzi P, D'Amico M, De Lorenzo V, Moscara L (2023). COVID-19 Vaccine Knowledge, Attitude, Acceptance and Hesitancy among Pregnancy and Breastfeeding: Systematic Review of Hospital-Based Studies. Vaccines.

[13] Centers for Disease Control (CDC), Family Health International (FHI), World Health Organisation (WHO) (1994). An epidemiological approach to reproductive health.

[14] Healy CM, Rench MA, Montesinos DP, Ng N, Swaim LS (2015). Knowledge and attitiudes of pregnant women and their providers towards recommendations for immunization during pregnancy. Vaccine.

[15] Kaliyaperumal KI (2004). Guideline for conducting a knowledge, attitude and practice (KAP) study. AECS illumination.

[16] Rasmussen SA, Kelley CF, Horton JP, Jamieson DJ (2021). Coronavirus disease 2019 (COVID-19) vaccines and pregnancy: what obstetricians need to know. Obstet Gynecol.

[17] Nassar AH, Visser GH, Nicholson WK, Ramasauskaite D, Kim YH, Barnea ER (2021). FIGO Statement: Vaccination in pregnancy. Int J Gynecol Obstet.

[18] Burgess C, Gasse F, Steinglass R, Yakubu A, Raza AA, Johansen K (2017). Eliminating maternal and neonatal tetanus and closing the immunity gap. Lancet.

[19] Ridpath AD, Scobie HM, Shibeshi ME, Yakubu A, Zulu F, Raza AA (2017). Progress towards achieving and maintaining maternal and neonatal tetanus elimination in the African region. Pan Afr Med J.

[20] Simpson CR, Lone NI, Kavanagh K, Robertson C, McMenamin J, Von Wissmann B (2017). Evaluating the effectiveness, impact and safety of live attenuated and seasonal inactivated influenza vaccination: protocol for the Seasonal Influenza Vaccination Effectiveness II (SIVE II) study. BMJ Open.

[21] Debellut F, Hendrix N, Ortiz JR, Lambach P, Neuzil KM, Bhat N (2018). Forecasting demand for maternal influenza immunization in low- and lower-middle-income countries. PLoS One.

[22] Shafiee A, Kohandel Gargari O, Teymouri Athar MM, Fathi H, Ghaemi M, Mozhgani SH (2023). COVID-19 vaccination during pregnancy: a systematic review and meta-analysis. BMC Pregnancy Childbirth.

[23] Centers for Disease Control and Prevention (2022). COVID-19 vaccines while pregnant or breastfeeding.

[24] World Health Organisation (2023). Weekly epidemiological record.

[25] Citu IM, Citu C, Gorun F (2022). Determinants of COVID-19 Vaccination Hesitancy among Romanian Pregnant Women. Vaccines.

[26] Skirrow H, Barnett S, Bell S, Riaposova S, Mounier-Jack S, Kampmann B (2022). Women's views on accepting COVID-19 vaccination during and after pregnancy, and for their babies: a multi-methods study in the UK. BMC Pregnancy Childbirth.

[27] Ramlawi S, Muldoon KA, Dunn SI, Murphy MS, Dingwall-Harvey AL, Rennicks White R (2022). Worries, beliefs and factors influencing perinatal COVID-19 vaccination: a cross-sectional survey of preconception, pregnant and lactating individuals. BMC Public Health.

[28] Song Y, Zhang T, Chen L, Yi B, Hao X, Zhou S (2017). Increasing seasonal influenza vaccination among high risk groups in China: Do community healthcare workers have a role to play?. Vaccine.

[29] Licata F, Pelullo CP, Della Polla G, Citrino EA, Bianco A (2023). Immunization during pregnancy: do healthcare workers recommend vaccination against influenza?. Front Public Health.

[30] Licata F, Romeo M, Di Gennaro G, Citrino EA, Bianco A (2023). Pertussis immunization during pregnancy: results of a cross-sectional study among Italian healthcare workers. Front Public Health.

[31] Mazzilli S, Tavoschi L, Lopalco PL (2021). Knowledge, attitudes and practices concerning pertussis maternal immunization in a sample of Italian gynaecologists. Hum Vaccin Immunother.

